# Genomics of *Serratia marcescens* Isolates Causing Outbreaks in the Same Pediatric Unit 47 Years Apart: Position in an Updated Phylogeny of the Species

**DOI:** 10.3389/fmicb.2020.00451

**Published:** 2020-03-31

**Authors:** Claudia Saralegui, Manuel Ponce-Alonso, Blanca Pérez-Viso, Laura Moles Alegre, Esperanza Escribano, Fernando Lázaro-Perona, Val F. Lanza, Miguel Sáenz de Pipaón, Juan Miguel Rodríguez, Fernando Baquero, Rosa del Campo

**Affiliations:** ^1^Servicio de Microbiología, Hospital Universitario Ramón y Cajal and Instituto Ramón y Cajal de Investigación Sanitaria, Madrid, Spain; ^2^Red Española de Investigación en Patología Infecciosa, Madrid, Spain; ^3^Unidad de Esclerosis Múltiple, Instituto de Investigación Sanitaria Biodonostia, Donostia-San Sebastián, Spain; ^4^Servicio de Neonatología, Hospital Universitario La Paz, and Universidad Autónoma de Madrid, Madrid, Spain; ^5^Servicio de Microbiología, Hospital Universitario La Paz, Madrid, Spain; ^6^Unidad de Bioinformática del IRYCIS, Madrid, Spain; ^7^Centro de Investigación Biomédica en Red de Epidemiología y Salud Pública, Madrid, Spain; ^8^Departamento de Nutrición y Ciencia de los Alimentos, Universidad Complutense de Madrid, Madrid, Spain

**Keywords:** *Serratia marcescens*, phylogeny, resistome, antibiotic susceptibility, nosocomial outbreak

## Abstract

The first documented nosocomial outbreak caused by *Serratia marcescens* in Spain occurred in 1969 at the neonatal intensive care unit (NICU) of the tertiary La Paz Children’s Hospital in Madrid, Spain, and based on the available phenotyping techniques at this time, it was considered as a monoclonal outbreak. Only 47 years later, another *S. marcescens* outbreak of an equivalent dimension occurred at the same NICU. The aim of the present study was to study isolates from these historical and contemporary outbreaks by phenotypic analysis and whole-genome sequencing techniques and to position these strains along with 444 publicly available *S. marcescens* genomes, separately comparing core genome and accessory genome contents. Clades inferred by both approaches showed high correlation, indicating that core and accessory genomes seem to evolve in the same manner for *S. marcescens.* Nine *S. marcescens* clusters were identified, and isolates were grouped in two of them according to sampling year. One exception was isolate 13F-69, the most genetically distant strain, located in a different cluster. Categorical functions in the annotated accessory genes of both collections were preserved among all isolates. No significant differences in frequency of insertion sequences in historical (0.18–0.20)—excluding the outlier strain—versus contemporary isolates (0.11–0.19) were found despite the expected resting effect. The most dissimilar isolate, 13F-69, contains a highly preserved plasmid previously described in *Bordetella bronchiseptica.* This strain exhibited a few antibiotic resistance genes not resulting in a resistant phenotype, suggesting the value of gene down expression in adaptation to long-term starvation.

## Introduction

*Serratia marcescens* is a ubiquitous environmental microorganism, but also a relevant nosocomial pathogen able to cause a broad spectrum of infections, particularly in neonates (Zingg et al., 2008). The first scientific reports involving *S. marcescens* in human infections occurred in the second half of the 20th century. Today, the main concerns regarding this opportunistic pathogen are its capability to spread in the hospital environment, to cause outbreaks, and its potential for expressing and disseminating antibiotic resistance, combining intrinsic mechanisms and acquired antimicrobial genes (Mahlen, 2011).

The first documented *S. marcescens* outbreak affecting pediatric patients in Spain occurred in 1969 at the neonatal intensive care unit (NICU) of La Paz Children’s Hospital, the main pediatric university hospital in Madrid. The outbreak lasted, with oscillations, until late 1974 (>5 years), with a high incidence of *S. marcescens* bacteremia (9.16% of all early neonatal sepsis during the period), frequently associated with gut colonization during the first 3 days of life (12.5% of 120 children tested) (Baquero et al., 1969). However, the microorganism had been found not to have colonized the mothers’ vaginal cavity (37 tested) (Baquero et al., 1977). The recovered isolates were considered as a single clone causing a monoclonal outbreak according to the available bacterial typing techniques, based on phenotypic tests that included biochemical reactions and antimicrobial susceptibility testing.

Forty-seven years later (2016), in the same NICU and after several intermittent mild episodes, a new severe *S. marcescens* outbreak was declared with an extremely high incidence of bacteremia, also preceded by gut *S. marcescens* enrichment (Escribano et al., 2019). The environmental surveillance of the affected NICU did not obtain conclusive results, and bacterial typing with molecular tools demonstrated the coexistence of several genetic lineages involved in this outbreak (Redondo-Bravo et al., 2019). During the interval between the first and second outbreaks, *S. marcescens* was isolated from patients but without causing outbreaks of comparable magnitude. The aim of the present study was to examine the genomes of *S. marcescens* isolates causing historical and contemporary prolonged outbreaks that occurred in the same NICU but separated by almost half a century (from 1969 to 2016). A new, updated phylogeny of the species allowed to position old and contemporary outbreak isolates in a broader evolutionary perspective.

## Materials and Methods

### Sample Collection and Processing

Outbreak-causing *S. marcescens* isolates were obtained from blood and/or fecal samples from preterm neonates admitted to the NICU of Hospital La Paz in Madrid, Spain, and were grouped into historical (21 isolates from 1969) and contemporary (five isolates from 2016) collections. The contemporary outbreak from which contemporary collection belonged to started in December 2016 and ended in March 2017. The historical isolates were conserved at room temperature as stabs (short slant) in 4 ml brain–heart infusion (BHI) agar in screw-threaded small neutral glass vials (1 cm wide, 5 cm high), tightly sealed with parafilm at room temperature, and had not been opened before 2016. To refresh the cultures, the agar was rehydrated for 2 h with BHI broth (Difco, United States), gently shaken, and seeded in Columbia blood agar plates. Ten out of 21 historical strains were able to grow overnight. Species identification was confirmed by matrix-assisted laser desorption ionization-time of flight mass spectrometry (Bruker, Germany), and all recovered isolates were re-stored at −80°C in sterilized half-skimmed milk.

### Pulse-Field Gel Electrophoresis Typing

Pulse-field gel electrophoresis (PFGE) was developed according to a previously described protocol (Villa et al., 2012) using the *Spe*I restriction enzyme. The band patterns were analyzed using BioNumerics software^1^, and the results representation was built based on Dice coefficients and the unweighted pair group method with arithmetic mean algorithm.

### Whole-Genome Sequencing, Assembly, and Annotation

Eight isolates (four from each period) were selected according to PFGE and submitted to whole-genome sequencing (WGS). DNA was obtained using the QIAamp kit (QiaAMP, Germany), determining their concentration and quality by Qubit fluorometer. DNA samples were sent to the Complutense University of Madrid for library preparation using the Nextera XT kit (Illumina, Inc., United States). Paired-end sequencing was performed using a MiSeq system (2 × 150 pb) (Illumina). Nullarbor bioinformatics pipeline was used for sequence analyses (v2; Seemann, T.)^2^. This tool includes Trimmomatic v0.38 software for trimming and quality filtering of reads (Bolger et al., 2014), Kraken software v1.0 (Wood and Salzberg, 2014) and Centrifuge software v1.0.4 (Kim et al., 2016) for species identification, SPAdes v3.12.0 software for *de novo* assembly (Bankevich et al., 2012), Prokka software v1.13 for annotation (Seemann, 2014), Abricate software v0.8 8 (Seemann, T.)^3^ using the Resfinder database for resistome identification, and Snippy software v4.4.1 (Seemann, T.)^4^ and Roary software v3.12.0 (Page et al., 2015) for core genome and pangenome calculation. The Phandango web-based tool (Hadfield et al., 2017) was used for pangenome visualization and plotting. PLACNETw free-access software (Vielva et al., 2017) was used for detection and graphical reconstruction of contigs harbored by putative plasmids (containing relaxases and/or replication initiator proteins), which were subsequently confirmed by the basic local alignment search tool. The Phaster web-based tool (Arndt et al., 2016) was used for prophage annotation. Insertion sequences (ISs) were annotated using ISEScan software (Xie and Tang, 2017).

### Phylogenetic Analysis of Historical and Contemporary Strains by Single-Nucleotide Polymorphism

A single-nucleotide polymorphism (SNP) calling “all against all” approach was applied to calculate how many SNPs were present between each pair of isolates. Subsequently, the SNP calling approach was used to evaluate the phylogenetic relationship among eight strains, both contemporary and historical ones. First, each genome was mapped against the reference genome (O1-16, accession number QYRU00000000) by Snippy-core. Next, SNP variant calling was performed (Snippy v4.0-dev2) on the mapped sequences, and the SNP’s phylogeny was inferred (FastTree v2.1.10). All steps were performed using Nullarbor bioinformatics pipeline.

### Phylogenetic Analysis of the Global *S. marcescens* Population by Core Genome Multilocus Sequence Typing

We built a core genome multilocus sequence typing (cgMLST) scheme for phylogenetic comparison among eight contemporary or historical strains and 444 *S. marcescens* genomes assembled as contigs, scaffolds, or complete genomes that were available in the RefSeq National Center for Biotechnology Information (NCBI) database^5^ (Supplementary Data). We used the KRAKEN toolkit and all available filters of the NCBI web tool to only include genomes robustly assigned to *Serratia* and exclude partial and anomalous genomes. Likewise, we only considered contigs of >500 bp. First, a pangenome allele database was set using all selected genomes (*n* = 452). Then, we built one MLST tree from the pangenome allele database, selecting allelic variations only present in the core genome (cgMLST), set at >95% of occurrence. All analyses were performed using the cano-wgMLST_BacCompare web-based tool (Liu et al., 2019). Trees were edited using the iTOL v4.4.2 web-based tool (Letunic and Bork, 2019).

### Accessory Genome Functional Analysis

We compared the accessory genome of the contemporary or historical outbreak isolates and all *S. marcescens* annotated genomes from the previously mentioned RefSeq NCBI database using AcCNET v1.2 software. Ward’s minimum variance hierarchical clustering method was applied to cluster genomes according to their predicted annotated proteome (Lanza et al., 2017). Only significant proteins robustly assigned to each cluster (>70% per cluster frequency, <70% per total frequency, and <0.001 adjusted *p*-value) were taken into account for further comparisons. The Clusters of Orthologous Groups of proteins (COGs) database was used to assign functional categories to the predicted proteome content. RStudio software v.1.2.5001 and Gephi v.0.9.2 were used for statistical analysis and plotting.

### Antimicrobial Susceptibility Testing

The MicroScan WalkAway automated system (Beckman Coulter, United States) was used to determine the minimal inhibitory concentration (MIC) values. EUCAST criteria for each antimicrobial were used to define the susceptible, intermediate, and resistant isolates^6^.

## Results

### *Serratia marcescens* Pulse-Field Gel Electrophoresis Typing and Whole-Genome Sequencing

**FIGURE 1:**
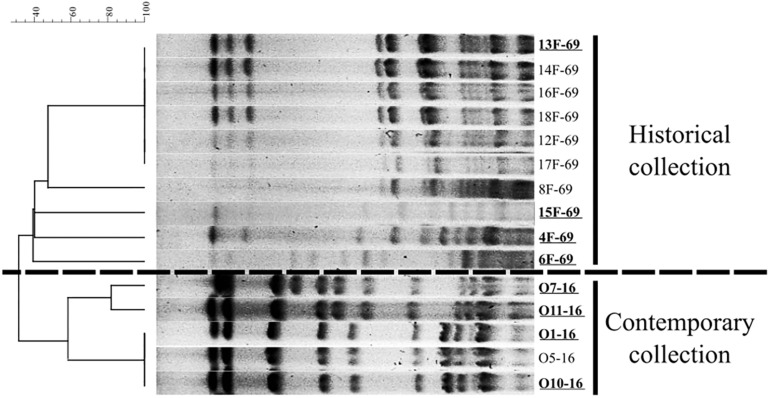
Dendrogram based on Dice’s coefficient showing the representative pulse-field gel electrophoresis (PFGE) pattern of the historical (10 out of 21 isolates) and the contemporary *Serratia marcescens* collections studied.

Ten out of 21 isolates from the historical collection were recovered after 24 h of overnight incubation and further analyzed. These historical strains were grouped in five PFGE patterns, and the five contemporary isolates were grouped into three pulsotypes (Figure 1). Four representative isolates from each period were finally submitted to WGS analysis (*n* = 8 strains). Data regarding isolation date and source of selected strains are included in Table 1. The relevant data of WGS throughput are summarized in Table 2, including accession numbers. The assembled genomes were deposited in the GenBank database and linked to BioProject PRJNA510235. The 13F-69 strain presented the lowest GC% content and the highest number of contigs.

**TABLE 1:** Isolation date and source of the eight *Serratia marcescens* studied isolates.

**Collection**	**Isolates**	**Isolation Date**	**Isolation Source**
**Historical**	4F-69	4/11/1969	Gut
	6F-69	6/11/1969	Gut
	13F-69	13/11/1969	Gut
	15F-69	15/11/1969	Gut
**Contemporary**	O1-16	21/11/2016	Gut
	O7-16	28/11/2016	Gut
	O10-16	8/12/2016	Gut
	O11-16	16/11/2016	Gut

**TABLE 2:** Sequencing information of the eight Serratia marcescens bacterial genomes compared in the study from Nullarbor pipeline report.

**Collection**	**Isolates**	**Accession Number**	**Contigs**	**Reads**	**Size (bp)**	**GC (%)**	**Seq. Depth**	**N50**
**Historical**	4F-69	QZVX00000000	20	2,374,810	5,035,544	59.6	63	2,820,768
	6F-69	RAIC00000000	23	1,394,190	5,095,011	59.6	37	518,765
	13F-69	SNQH00000000	65	2,457,194	5,279,779	59.1	66	163,753
	15F-69	RAID00000000	22	1,840,822	5,238,206	59.5	49	2,937,699
**Contemporary**	O1-16	QYRU00000000	26	3,232,838	5,102,926	59.7	87	600,674
	O7-16	QYRV00000000	21	2,215,906	5,127,038	59.7	59	3,175,227
	O10-16	QYSA00000000	22	1,950,698	5,105,131	59.7	52	600,674
	O11-16	QYSB00000000	23	1,925,266	5,057,421	59.8	51	580,213

*N50, minimum contig length needed to cover 50% of the genome; GC, percentage of guanine-cytosine content.*

### Functional Annotation, Plasmids, and Prophage Identification

**FIGURE 2:**
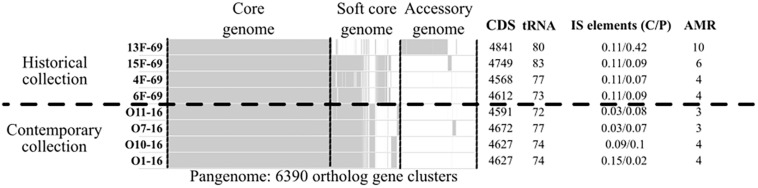
Nullarbor report of the pangenome of the eight *Serratia marcescens* studied strains, including as additional data the frequency of completely (C) or partially (P) present insertion sequences (ISs), and the number of antimicrobial resistance genes (AMR). Core genome is constituted by genes present in >99% of strains. Soft core genome is constituted by genes present in 99-15% of strains. Accessory genome is constituted by genes present in <15% of strains. CDS, protein-coding sequences.

**FIGURE 3:**
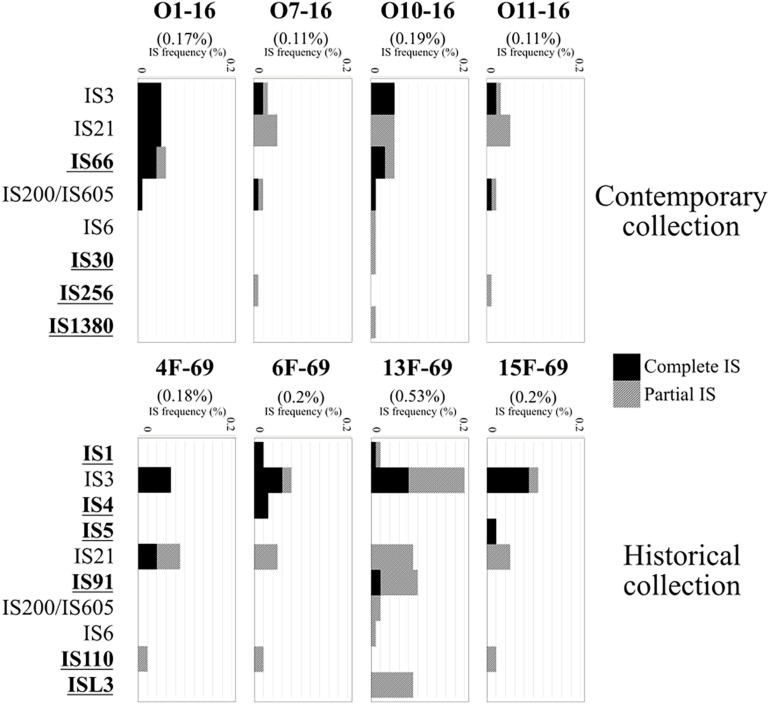
Insertion sequences (ISs) detected in the eight *Serratia marcescens* studied isolates and their frequency in the annotated genomes, represented as IS bp/total genome bp (%). Total IS frequency in each genome is expressed in parentheses. ISEScan output included integral ISs and partially present ISs (black and gray bars, respectively). Bold, underlined IS labels were found only in one collection but not in the other.

A total of 6,390 different clusters of orthologous genes constituted the pangenome of the eight studied outbreak isolates (four isolates from the historical and four isolates from the contemporary collection). The core genome (genes present in >99% of strains as established by the Nullarbor pipeline) constituted 3,642 (57%) gene clusters, whereas 1,237 (19.4%) gene clusters corresponded to the accessory genome (genes present in <15% of strains). All isolates presented a similar number of protein-coding sequences (CDSs) and tRNA copies. A summary of the gene distribution and additional data are presented in Figure 2, whereas IS profiles are displayed in Figure 3.

The historical 13F-69 and 15F-69 isolates were remarkably different from the others (highest CDS, tRNA, and IS content), and all data indicated that numerous genetic exchange events had occurred in these strains. The contemporary isolates appeared to present higher genetic stability with slightly lower total IS content. From the 14 different IS families detected, eight were detected in a single isolate, five were present in two or three isolates, and only IS3, IS21, and IS200/IS605 appeared to be regularly represented. It is important to note that IS200/605 was only detected in its complete form in the four contemporary isolates, although a partial IS200/605 sequence was also observed in the 13F-69 strain. Mann–Whitney U test for independent samples showed significant differences (*p* < 0.05) between historical and contemporary total IS percentage. Nevertheless, when the outlier 13F-69 was extracted from the comparison, no significant differences resulted.

**FIGURE 4:**
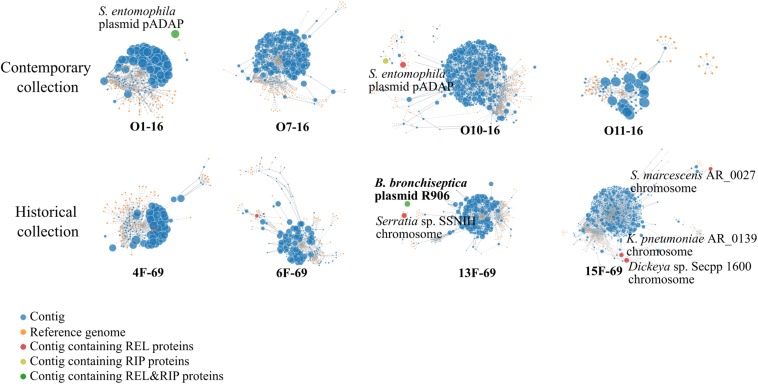
PlacnetW output reflecting the plasmid sequences detected. All contigs from each genome are represented as blue nodes connected by continued lines. Yellow nodes represent reference genomes from the NCBI BLAST database. Discontinued lines link each contig to their closest reference genome/s. Finally, each genome appears represented by all its contigs displayed around their closest reference genome identity. Contigs containing plasmid sequences are highlighted in different colored nodes. REL, relaxase; RIP, replication initiation protein.

According to the PlacnetW report, incomplete plasmids were detected in the O10-16, 13F-69, and 15F-69 strains (Figure 4 and Table 3). The only complete plasmid, R906, corresponded to the one previously reported in *Bordetella bronchiseptica* and was found in the historical 13F-69 isolate. This plasmid carried genes encoding antibiotic resistance [*oxa*-2, *sul-*1, *qac*EΔ1, *aph*(3′)-II, and multidrug transporters *mep*C and *mdt*E], nickel–cobalt resistance (*cnr*A), quaternary ammonium resistance (*sug*E), type IV secretion system proteins, toxins (*fit*B), and iron transporters. Other fragments identified as putative plasmids shared sequences with extra-chromosomal elements of *Serratia entomophila* and *Serratia* spp. or with the chromosome of *S. marcescens*, *Klebsiella pneumoniae*, *Serratia* sp., or *Dickeya* sp. (Figure 4). Regarding lysogenic phages present in the studied isolates, a total of five complete prophages were identified, all belonging to the *Caudovirales* order (Table 3).

**TABLE 3:** Plasmid and prophage sequences annotated by PlacnetW and Phaster software, respectively.

**Isolate**	**Plasmids and prophage sequences**	**Similarity^a^**	**Contigs^b^**	**Size(bp)**	**CDS**
13F-69	*Serratia* sp. SSNIH^c^	50/99.7	1	119,213	115
	***B. bronchiseptica* R906 plasmid**	**99/99.6**	**1**	**57,271**	**64**
	*Siphoviridae* phage	100	–	17,500	18
15F-69	*S. marcescens* AR_0027^c^	30/98	1	53,451	52
	*Dickeya* sp. Secpp 1600^c^	41/98	1	26,298	12
	*K. pneumoniae* AR_0139^c^	80/96.5	1	83,980	92
O7-16	*Siphoviridae* phage	110	–	43,600	61
	*Myoviridae* phage	150	–	34,400	40
	*Myoviridae* phage	110	–	20,400	25
O1-16	pADAP plasmid	55/76.9	1	83,980	92
O10-16	pADAP plasmid	64.3/75.2	2	66,877	85
O11-16	*Myoviridae* phage	150	–	34,400	40

*The unique complete plasmid was highlighted in bold. CDS, protein-coding sequence. ^*a*^Identity score of phages (minimum threshold = 60) and plasmids (expressed as query cover/% identity from BLAST report). ^*b*^Number of contigs separately plotted in PlacnetW that matched with the same BLAST identification. ^*c*^Contigs plotted as putative plasmids by PlacnetW software which sequences matched with fragments of bacterial chromosomes.*

### Antibiotic-Resistant Genes and Susceptibility

Both in the historical and contemporary outbreaks, the majority of genomes contained three or four antibiotic resistance genes, except the historical 13F-69 and 15F-69 isolates, which carried 10 and six genes, respectively (Table 4). Interestingly, 13F-69 remained phenotypically susceptible to almost all tested antibiotics, excluding those affected by *S. marcescens* constitutive resistance (Table 5). Only two isolates, one from each period, exhibited resistance to broad-spectrum beta-lactam antibiotics, presumably due to mechanisms involving *amp*C de-repression.

**TABLE 4:** Resistome derived from WGS analysis of the eight *Serratia marcescens* isolates of the study and major antibiotic classes which can be potential targets of the products the resistance genes encode.

**Antibiotic Class/Resistome**	**O1-16**	**O7-16**	**O10-16**	**O11-16**	**4F-69**	**6F-69**	**13F-69**	**15F-69**
**Beta-lactams**								
*bla*OXA-2							✓ (p)	
*bla*SRT-1	✓		✓				✓	
*bla*SRT-2		✓		✓	✓	✓		✓
**Aminoglycosides**								
*aac*(6’)		✓	✓	✓	✓	✓	✓	✓
*aph*(3”)-Ib							✓ (p)	✓
*aph*(6)-Id							✓	✓
**Quinolones**								
*qnr*B75							✓	
*qnr*E1	✓		✓		✓	✓		✓
**Sulfonamide**								
*sul-*1							✓ (p)	
**Multidrugs (oqx, chl)**								
*oqx*B24							✓	
*oqx*B9	✓	✓	✓	✓	✓	✓		✓
**QUATS**								
*qacEΔ1*							✓ (p)	
**Tetracycline**								
*tet*(41)							✓	

*CHL, chloramphenicol; OQX, olaquindox; WGS, whole-genome sequencing; (p), gene located in plasmid.*

**TABLE 5:** Observed minimal inhibitory concentrations (MICs) (mg/L) or zone of inhibition diameter (ZD) (mm) of each antibiotic for the eight studied *Serratia marcescens* strains.

**Antibiotics and**	**Available**	**Contemporary collection**				**Historical collection**			
**Resistance genes**	**since**	**MICs (mg/L) or ZD (mm)**				**MICs (mg/L) or ZD (mm)**			
		**O1-16**	**O7-16**	**O10-16**	**O11-16**	**4F-69**	**6F-69**	**13F-69**	**15F-69**
**Beta-lactams**									
Ampicillin	1961	**>16**	**>16**	**>16**	**>16**	**>16**	**>16**	**>16**	**>16**
Amoxicillin–Clavulanate	1972	**>16/8**	**>16/8**	**>16/8**	**>16/8**	**>16/8**	**>16/8**	**>16/8**	**>16/8**
Piperacillin–Tazobactam	1993	≤8	≤8	≤8	≤8	≤8	**>64**	≤8	≤8
Cefazolin	1971	**>16**	**>16**	**>16**	**>16**	**>16**	**>16**	**>16**	**>16**
Cefuroxime	1978	**>16**	**>16**	**>16**	**>16**	**>16**	**>16**	**>16**	**>16**
Cefoxitin	1972	16	**>16**	**>16**	16	16	**>16**	16	16
Ceftazidime	1984	≤1	≤1	**>16**	≤1	≤1	**>16**	≤1	≤1
Cefotaxime	1980	≤1	≤1	**>32**	≤1	≤1	**>32**	≤1	≤1
Cefepime	1994	≤1	≤1	**>16**	≤1	≤1	**8**	≤1	≤1
Aztreonam	1986	≤1	≤1	**>16**	≤1	≤1	**>16**	≤1	≤1
Imipenem	1977	≤1	≤1	≤1	≤1	≤1	≤1	≤1	≤1
Ertapenem	2001	≤0.5	≤0.5	**>4**	≤0.5	≤0.5	≤0.5	≤0.5	≤0.5
**Aminoglycosides**									
Gentamicin	1963	≤2	≤2	**>8**	≤2	≤2	≤2	≤2	≤2
Tobramycin	1997	≤2	≤2	**>8**	≤2	≤2	4	4	≤2
Amikacin	1976	≤8	≤8	**>32**	≤8	≤8	≤8	≤8	≤8
Streptomycin*	1943	15	15	**11**	16	**13**	17	**0**	**9**
**Ciprofloxacin**	1987	≤0.5	≤0.5	**1**	≤0.5	≤0.5	≤0.5	≤0.5	≤0.5
**Nalidixic acid**	1965	≤16	≤16	>16	≤16	≤16	≤16	≤16	≤16
**TMP/SMX**	1968-78	≤2/38	≤2/38	≤2/38	≤2/38	≤2/38	≤2/38	≤2/38	≤2/38
Tetracycline*	1953	**14**	**12**	**15**	**13**	21	21	**0**	**12**

*MIC values or zone diameters over the clinical cutoff (based on EUCAST 2019 criteria) are highlighted in bold, underlined numbers. NA, not available; TMP/SMX, trimethoprim/sulfamethoxazole. Asterisk, antibiotics tested only by disk diffusion test (25 μg for streptomycin and 30 μg for tetracycline).*

### Phylogenetic Analysis Within Contemporary and Historical Outbreak Strain Collections by the Single-Nucleotide Polymorphism Approach

**FIGURE 5:**
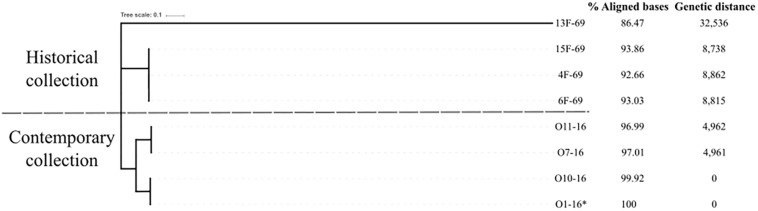
Phylogenetic tree based on single-nucleotide polymorphism (SNP) analysis of the mapped sequences of the eight *Serratia marcescens* studied strains using the O1-16 strain as reference (*). Genetic distance was obtained by dividing the number of SNPs present in each genome by the aligned megabases with the reference.

The “all against all” SNP matrix showed the isolates had a certain degree of genetic polymorphism within each outbreak. In the contemporary outbreak, there were two clones, one represented by O1-16 and O10-16 and another represented by O7-16 and O11-16, whereas more SNPs were identified within isolates from the historical collection (Table 6). Then, one isolate from the contemporary collection (O1-16) was taken as the reference genome for constructing a phylogenetic tree based on SNP core distance. A total of 173,766 SNPs were found in the mapped regions of the eight studied isolates with O1-16 as a reference. Phylogenetic distances from reference strain O1-16 were 0–4,962 in the four strains of contemporary collection and 8,738–8,862 in three out of four in the historical collection. The outlier strain 13F-69 had a value of 32,536 and constituted a separate distant branch (Figure 5).

**TABLE 6:** SNP matrix including all SNPs present between pairs of isolates.

	**O1-16**	**O7-16**	**O10-16**	**O11-16**	**4F-69**	**6F-69**	**13F-69**	**15F-69**
O1-16	0	24,557	0	24,524	41,900	41,895	141,431	41,916
O7-16	24,554	0	24,555	1	42,382	42,366	141,588	42,403
O10-16	0	24,562	0	24,516	41,898	41,878	141,320	41,914
O11-16	24,562	0	24,556	0	42,392	42,371	141,574	42,405
4F-69	41,864	42,376	41,865	42,340	0	37	141,842	53
6F-69	41,779	42,295	41,788	42,239	36	0	141,382	23
13F-69	143,127	143,429	143,332	143,324	143,904	143,990	0	143,868
15F-69	41,821	42,330	41,817	42,287	49	19	141,762	0

*Isolates in columns are the ones to map the SNP content to. SNP, single-nucleotide polymorphism.*

### *Serratia marcescens* Core Genome Multilocus Sequence Typing Scheme

**FIGURE 6:**
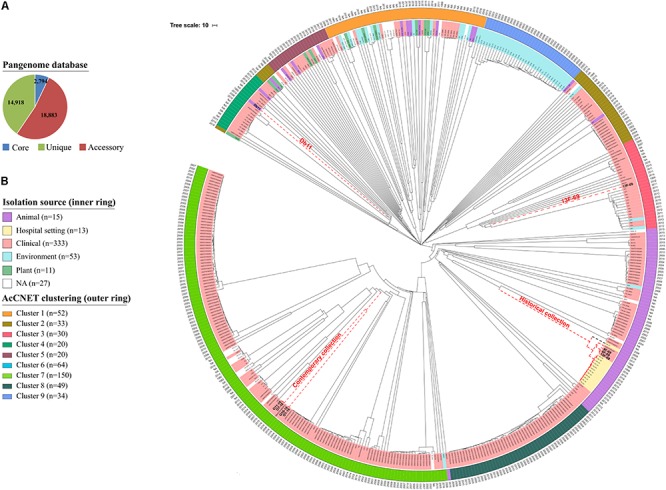
Phylogenetic analysis of 452 *Serratia marcescens* genomes. **(A)** Distribution of the total pangenome after core genome multilocus sequence typing (cgMLST) analysis in core (genes present it at least 95% of the isolates; 7.6%), accessory (genes present in >2 isolates and less than in 95%; 51.6%), and unique genes (genes exclusive of a single isolate; 40.8%). **(B)** Phylogenetic tree inferred from cgMLST approach, including metadata of source (colors) and year (text). Strains from our collections and the reference genome for *S. marcescens* species (Db11 strain, assembly accession GCA_000513215.1) are highlighted in red dashed nodes. External circle highlights clusters inferred by AcCNET analysis of accessory genome.

The aim of this analysis was to compare all eight historical or contemporary isolates along with 444 complete genomes in the *S. marcescens* database by constructing a cgMLST scheme. This analysis showed that the *S. marcescens* pangenome comprised a total of 36,595 genes, 7.6% of which were shared by >95% of the 452 isolates (core genome) (Figure 6A). Phylogeny was inferred based on allelic variations across this core genome (Figure 6B). Contemporary isolates clustered together and were closely related to a human clinical specimen obtained from blood in 2006 in the United Kingdom (strain: 2880STDY5682913, BioProject: PRJEB5065). This 2006 strain showed 26,242 nucleotide variants when compared to the O1-16 strain and 703 variants when compared to the O7-16 strain. Historical isolates were located in a separate branch close to a strain from the same BioProject collected in 2011 (strain: 2880STDY5683020). Again, the historical 13F-69 isolate was the most distant and was related to clinical strains from Brazil (BioProject reference: PRJNA420811). It is important to note that most of the annotated public genomes have a clinical or hospital origin (76.5%), whereas other sources [environmental (11.7%), animal (3.3%), plant (2.4%), and miscellaneous (6%)] are considerably less represented.

### Accessory Genome Comparison Using Publicly Available Databases

**FIGURE 7:**
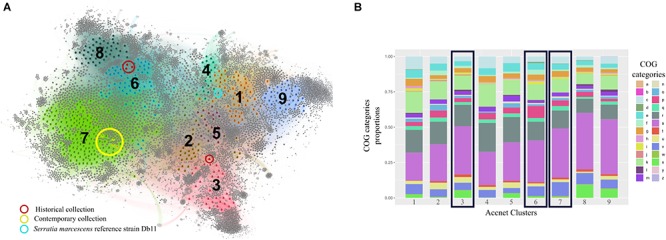
Clustering and analysis of the categorical functions of the accessory genome of the studied *Serratia marcescens* isolates (*n* = 8) and the 444 publicly available genomes by AcCNET software. **(A)** AcCNET pangenome network with detected clusters (*n* = 9) highlighted by colors and numbers. Colored and gray dots correspond to genomes and annotated proteins respectively and are linked by edges. Strains from our collections and the reference genome for *S. marcescens* species (Db11 strain, assembly accession GCA_000513215.1) are highlighted. **(B)** Accessory genome functional analysis of the nine detected clusters based on COGs proportions. Black rectangles depict the clusters where strains from our collections belong to. a, RNA processing and modification; b, chromatin structure and dynamics; c, energy production and conversion; d, cell cycle, control, mitosis; e, amino acid metabolism and transport; f, nucleotide metabolism and transport; g, carbohydrate metabolism and transport; h, coenzyme metabolism; i, lipid metabolism; j, translation; k, transcription; l, replication and repair; m, cell wall/membrane/envelope biogenesis; n, cell motility; o, post-translational modification, protein turnover, chaperon functions; p, inorganic ion transport and metabolism; q, secondary structure; r, general function prediction only; s, function unknown; t, signal transduction; u, intracellular trafficking and secretion; v, defense mechanisms; W, extracellular structures; x, mobilome, prophages, and transposons-related proteins; y, nuclear structure; z, cytoskeleton.

The content of the accessory genome of the eight studied outbreak isolates was compared with the 444 previously annotated *S. marcescens* genomes deposited in public databases using AcCNET software. This analysis differentiated all genomes into nine clusters according to their predicted accessory proteome content. Historical strains were located in cluster 6, with the exception of isolate 13F-69, which was included in cluster 3, and contemporary strains were located in cluster 7 (Figure 7A). This distribution regarding the accessory genome was compared to that inferred by cgMLST analysis in Figure 6B, and all strains were clustered in a similar manner. No outbreak isolates were found in any of the other seven clusters of *S. marcescens.*

The hierarchical clustering method used in this work minimizes the total within-cluster variance, and the nine resulting clusters were the most homogeneous possible. Cluster 7 was the most frequently represented among the *S. marcescens* genome collection, grouping 33.2% of the total isolates, followed by cluster 6 (14.2%) and cluster 1 (11.5%). The other clusters are less represented probably because they include infrequent accessory genes that can only be found sporadically. We cannot rule out the possibility that overrepresentation of cluster 7 could have been influenced by sampling bias (see section “*Discussion”*).

The functional category of each accessory gene was assigned using the COGs database. Interestingly, the genes of the nine clusters had analogous functions, even though the genetic content was different (Figure 7B). The top significant proteins of the accessory genome separating clusters 3, 6, and 7 from the rest are summarized in Table 7.

**TABLE 7:** Top relevant proteins of the accessory genome that significantly explained the discrimination of each of the three clusters to which the eight *Serratia marcescens* studied strains belong to, with respect to the overall population, analyzed by AcCNET software.

**COGs Categories**	**Cluster 3 (13F-69 isolate)**	**Cluster 6 (Historical collection)**	**Cluster 7 (Contemporary collection)**
**C**	Gfo/Idh/MocA family [*Enterobacterales*] Taurine dioxygenase TauD [*Serratia*] Tartrate dehydrogenase [*Enterobacterales*]	CbbBc protein Nitrite reductase	NADP-oxidoreductase [*Enterobacterales*] Short-chain reductase [*Enterobacterales*] (2Fe–2S)-binding protein Electron transport complex
**D**	NA	Cell division protein Fic/DedD	Cell division protein Fic
**F**	(S)-ureidoglycine aminohydrolase [*Enterobacterales*] Ureidoglycolate dehydrogenase [*Enterobacterales*]	Pyridine nucleotide-disulfide oxidoreductase [*Serratia*] (S)-ureidoglycine aminohydrolase [*Enterobacterales*] Ureidoglycolate dehydrogenase [*Enterobacterales*] 5′-nucleotidase, lipoprotein e(P4) family	Pyridine nucleotide-disulfide oxidoreductase [*Serratia*] Tyrosine-protein kinase Wzc [*Serratia*]
**J**	Methylase [*Enterobacterales*] tRNA-binding protein [*Enterobacterales*] tRNA(fMet) endonuclease [*Serratia*]	NA	NA
**L**	Recombinase family protein Antirestriction protein Conjugative transfer relaxase DNA-damage inducible protein J [*Serratia*]	DNA polymerase III [*Enterobacterales*]	NA
**P**	TonB-dependent receptor Ions translocating ATPase [*Serratia*] Siderophore biosynthesis protein [*Serratia*]	TonB-dependent receptor Ions translocating ATPase [*Serratia*] Multicopper oxidase CueO	TonB-dependent receptor Ions translocating ATPase [*Serratia*] Multicopper oxidase CueO Ferrichrome porin FhuA
**U**	Hemolysin secretion protein Type VI secretion system [*Serratia*] Type IV secretion system [*Enterobacterales*] Microcin H47 secretion protein [*Serratia*] LysE family translocator [*Serratia*]	Hemolysin secretion protein Type II secretion system HlyD family secretion protein	Hemolysin secretion protein Type II secretion system HlyD family secretion protein
**V**	Type 1 fimbrial protein Type II toxin-antitoxin system Colicin V biosynthesis protein Tetracycline efflux transporter Aminoglycoside transporter MBL fold metallo-hydrolase Stress protection protein MarC [*Serratia*] Tellurite-resistance protein TehA Exotoxin	Type 1 fimbrial protein Type II toxin-antitoxin system [*Serratia*] Multidrug efflux pump RND [*Serratia*] Toxin-activating lys-acyltransferase [*Serratia*] Antibiotic biosynthesis monooxygenase [*Enterobacterales*] Class A beta-lactamase-related serine hydrolase Addiction module antidote protein [*Enterobacterales*]	Type 1 fimbrial protein Type II toxin-antitoxin system [*Serratia*] Multidrug efflux pump RND [*Serratia*] Toxin-activating lys-acyltransferase [*Serratia*] Antibiotic biosynthesis monooxygenase [*Enterobacterales*] Exotoxin Filamentous hemagglutinin Antitoxin [*Serratia*]
**X**	Type-F conjugative transfer system [*Klebsiella*] IS110 transposase [*Proteobacteria*] IS200/IS605 transposase Holin [*Enterobacterales*] Plasmid replication initiator [*Enterobacterales*] IS5 transposase Competence protein TfoX	Killer protein [*Serratia*] Prevent-host-death protein [*Serratia*]	Transposase

*Note that some classes of proteins are shared among clusters. Some taxonomic ranges are included when the annotated proteins did not reach the category of *S. marcescens* species. COGs, Clusters of Orthologous Groups of proteins; NA, not applicable.*

## Discussion

Epidemiological studies combined with bacterial typing are essential for controlling nosocomial outbreaks, given they help to detect high-risk hospital-adapted clones, which have the potential to spread globally, and to improve local containment measures. Bacterial typing techniques have evolved over time—from phenotypic tests (biotypes, antibiotypes) in the 70s of the previous century to current molecular studies based on DNA, with a superior discrimination capacity, better interlaboratory reproducibility, and greater cost-effectiveness (Mellmann et al., 2017; Pérez-Losada et al., 2017). In this study, we had the opportunity to re-type by WGS a historical collection of *S. marcescens* causing a NICU outbreak. Those isolates had initially been classified as forming part of a monoclonal outbreak; however, we were able to distinguish the coexistence of two lineages by PFGE, and WGS demonstrated the apparent individuality of each one of the sequenced isolates. In addition, within the same NICU settings, we compared historical isolates with their contemporary counterparts trying to decipher whether there were common features that explained the successful transmission rate of these outbreak strains separated by 47 years.

The most frequently used typing techniques for relevant human-related pathogens are PFGE and MLST; however, for *S. marcescens*, the MLST scheme has not yet been implemented (Martineau et al., 2018). Currently, WGS is a much better option given it is cost-effective in comparison with MLST and it reveals the composition of the accessory genome. In addition, WGS allows (i) the detection of SNP mapping against a reference genome and (ii) a gene by gene-based comparative approach according to a previously constructed pangenome allele database (PGAdb) and further clustering of the genomes [whole genome multilocus sequence typing (wgMLST)] (Schürch et al., 2018). The dataset can also be curated to include only a set of shared genes, e.g., the core genome (cgMLST) given it has recently been used to distinguish *S. marcescens* hospital-adapted lineages from those of environmental origin (19) (Abreo and Altier, 2019). The SNP approach is more sensitive than cg/wgMLST analysis but lacks interlaboratory reproducibility due to the need to establish a concrete reference. Beyond epidemiological investigations, WGS is useful for assessing the genetic evolution of bacterial species, particularly when the accessory genome is analyzed, as it has recently been described for *Enterococcus faecalis* (León-Sampedro et al., 2019). New genomic and proteomic analysis tools as, for example, AcCNET, have significantly improved such analysis (Lanza et al., 2017).

In the present study, both SNP and cgMLST (taking a set of genes shared by >95% of the whole database) were performed, together with an accessory genome analysis. The pangenome composition obtained in our study included over 36,000 gene clusters. Probably wide pangenomes correspond to ubiquitous organisms submitted to many environmental conditions. In contrast, pangenomes from a bacterial organism with a high degree of habitat specialization are much more limited. The pangenome inferred from 50 *Streptococcus* genomes is considerably lower, 5,398 gene clusters (Contreras-Moreira and Vinuesa, 2013). The size of *Serratia* pangenome is closer to that of another environmental-promiscuous genus, *Vibrio*, composed of over 26,000 gene clusters in a study involving 32 genomes (Thompson et al., 2009). These findings reveal the high diversity of unique genes present in *S. marcescens*, most probably corresponding to genes present in mobile genetic elements, genomic islands, transposons, and prophages.

Isolates from both historical and recent collections were consistently grouped by sampling period, being the historical 13F-69 the most outstanding isolate in all analyses performed. Both the core genome and accessory genome analyses grouped both collections in a similar way (Figure 6B), a fact that has been previously described in similar scenarios (Moradigaravand et al., 2016; Abreo and Altier, 2019). This observation indicates that *S. marcescens* appears to evolve in the same direction for both core and accessory genes.

*Serratia marcescens* is particularly associated to NICU environment, being certain lineages more associated to sepsis than others (Moles et al., 2019). The coexistence of several clones in the same *S. marcescens* outbreak, as occurred in our historical collection, had previously been reported (Fleisch et al., 2002; Montagnani et al., 2015; Dawczynski et al., 2016). The cgMLST scheme revealed that most of our isolates share a common ancestor with most of the published clinical strains. However, a caveat of this analysis is the low representation of non-clinical isolates (<24% of the total) in the public genome databases. Comparing our isolates with the previously published genomes, the contemporary collection was located next to a multidrug-resistant nosocomial strain collected in the United Kingdom in 2006; the historical collection next to several strains collected from ICU surfaces in Pakistan in 2016; and finally, the 13F-69 isolate was grouped in a separate branch with nosocomial isolates from Brazil isolated in 2012. This analysis, to our knowledge, is the most extensive study of the phylogeny of *S. marcescens* to date; however, as previously mentioned, an important lack of isolates from non-hospital sources was observed.

The accessory genome determines the idiosyncrasy of each strain, but it appears to correlate with the core genome content throughout evolution, as previously observed (Moradigaravand et al., 2016). Each one of the nine clades that we were able to discriminate had a unique combination of accessory genes compared with the whole collection. On one hand, clusters 6 and 7 appeared to be more related to each other than to cluster 3, where 13F-69 isolate was located. Both clusters share proteins involved in defense mechanisms such as type II toxin/antitoxin systems and multidrug efflux pumps, and also in the cell cycle, intracellular trafficking and secretion, and inorganic ion transport and metabolism. Cluster 6 seems to be enriched in proteins involved in nucleotide metabolism and transport, such as aminohydrolases and dehydrogenases for anaerobic nitrogen utilization, whereas cluster 7 is enriched in proteins related to energy production and conversion, and they are more faithfully assigned to the taxonomic range of *S. marcescens* species. On the other hand, proteins in cluster 3 are more frequently assigned to upper taxonomic ranges such as phylum or order (*Proteobacteria, Enterobacterales*). This cluster is enriched in proteins related to translation processes, intracellular trafficking and secretion, and proteins that constituted the mobilome, prophages, and transposons-related proteins such as conjugative systems, transposases, and plasmid replication initiators. Overall, the accessory genome of our strains carried typical features that allowed them to survive in a clinical setting. In a previous publication, several efflux pumps had been detected in a collection of 32 *Serratia* genomes from various sources, with the clinical strains the ones carrying the highest number of efflux pumps and acquired resistance genes *via* horizontal gene transfer elements (Sandner-Miranda et al., 2018). Resistance genes coding for efflux pumps, as well as their regulatory elements, should be taken into consideration as influencing the success of antibiotic therapy.

A relevant finding was that the 13F-69 strain harbors a highly conserved plasmid previously described in *B. bronchiseptica*, a zoonotic pathogen of the respiratory airways. Plasmid exchange between phylogenetically distant species (in our case from Beta-*Proteobacteria* to Gamma-*Proteobacteria* taxa) is a rare event. Such transmission appears to have occurred despite distant natural niches, although the gut–lung axis or maybe a lung coinfection could justify a common nexus (Matthieu et al., 2018). In any case, the full preservation of the R906 plasmid after a rest period of 47 years is noteworthy. Of course, the origin of the plasmid was inferred from the available data, and we cannot rule out *Serratia* as its primary host. Finally, this plasmid contains the YafQ-DinJ toxin–antitoxin system, which could be involved in long-term plasmid maintenance. Four prophages were identified in the contemporary collection, whereas only one was found in the historical collection, a result probably related to current genome plasticity requirements (Shen et al., 2019).

Historical collections of bacteria provide us with relevant data from the pre-antibiotic period (Baker et al., 2014, 2015; Devault et al., 2014). Our historical isolates were collected from 1969 to 1970 during the antibiotics classic golden age, but they had not been exposed to newly discovered antimicrobials. However, these historical isolates carried genes coding for resistance to antibiotics and disinfectants that were commercialized years later. Although olaquindox is an antimicrobial agent only used since 1975 as a growth promoter for farm animals, the olaquindox resistance genes *oqx*10, *oqx*24, and *oqx*9 were consistently found in our historical isolates. This finding could possibly be explained by other functions that are provided by the multidrug efflux pumps encoded by *oqx* genes, including quinolone and benzalkonium chloride resistance (Hansen et al., 2007). Plasmid-mediated quinolone-resistance *qnr* genes were discovered in 2002 (Tran and Jacoby, 2002); however, *qnr*B75 and *qnr*E1 were already present in our historical isolates probably as the *qnr* function is not related primarily with antibiotic resistance (Hernández et al., 2011). Alternatively, they could have emerged under quinolone selection. In our days, quinolones or fluoroquinolones are not used in pediatrics. However, in 1969, at the time of the first *S. marcescens* outbreak, the first quinolone, nalidixic acid, was extensively used to control epidemic enteritis by enteropathogenic *Escherichia coli* at La Paz Children’s Hospital, which could have contributed to the selection of *qnr* genes in intestinal *Serratia* strains.

Intrinsic resistance in *Serratia* includes penicillins, macrolides, lincosamines, linezolid, glycopeptides, quinupristin-dalfopristin, and rifampin (Mahlen, 2011). Ampicillin and tetracycline resistance has typically been considered intrinsic, but that assumption could be due to the bias imposed by clinical strains, and a number of environmental strains could be susceptible (Ehrenkranz et al., 1980). Probably all *S. marcescens* strains harbor a chromosomal AmpC beta-lactamase, normally repressed. Mutants in AmpC and AmpD (restricting the function of AmpR, the AmpC repressor) result in a constitutive expression of AmpC, now able to deactivate third- and fourth-generation cephalosporins (Raimondi et al., 2001; Kaneko et al., 2005; Meini et al., 2018). This appears to have occurred in the historical strain 6F-69 and the contemporary strain O10-16, resistant to cefotaxime, ceftazidime, and cefepime. However, we were unable to detect mutational changes in the structural or known regulatory genes of the AmpC beta-lactamase (*ampC*, *ampD, ampE*, *ampG, ampR*, and their flanking sequences); this genotype–phenotype discrepancy has been previously observed in other species (Campbell et al., 1997) so that probably the complex regulation of AmpC production in *Serratia* should be further clarified in the future. Strain O10-16 was also resistant to carbapenems probably due to concurrence with porin mutations impairing antibiotic entry (Mammeri et al., 2008). In the 1970s, *S. marcescens* antimicrobial therapy was based mostly on carbenicillin and gentamicin, sometimes combined with fosfomycin (Baquero et al., 1977). However, the historical 6F-69 isolate exhibited resistance to cephalosporins that had not been discovered in 1969 probably because of the hospital exposure and selection of de-repressed mutants by cephaloridine, introduced in 1962. Likewise, gentamicin resistance was detected in the modern O7-16 isolate even though most *S. marcescens* isolates used to be susceptible due to the infrequent use of this antimicrobial (Moradigaravand et al., 2016).

Finally, it is relevant to discuss the potential influence of the prolonged rest period experienced by our “old” strains. In a classic publication (Naas et al., 1994), Werner Arber’s group compared a strain of *E. coli* K-12 (W3110) stabbed in 1960 with subclones recovered in 1965, 1972, and 1990 from small portions of the original stab, which was resealed immediately. Over these 30 years, the resting culture appeared to have developed a high degree of genetic diversity, which was an unexpected finding, considering the reduced rate of propagation during storage. The source of most of such variability was attributed to IS transposition (Naas et al., 1994, 1995), suggesting that the genome of resting bacteria is more dynamic than was long believed. In the present study, we found several ISs in our strains, with more diversity and a slightly higher proportion in the historical collection; however, major differences could not be established. This result could indicate less dynamism in resting strains of *S. marcescens* compared with those observed in *E. coli* (Naas et al., 1994). As mentioned before, WGS demonstrated the carriage in the resistome of the historical strain 13F-69 of several antibiotic-resistant genes, but from some of them, as *qnrB*, *qnrE*, acting on quinolones, or *tet*(41), encoding a tetracycline efflux pump, failed to produce the expected resistance phenotype. We cannot discard a decrease in expression as a strategy to reduce the fitness cost of the organisms under a very prolonged rest period to decrease fitness costs (Enne et al., 2006; Allen et al., 2017).

In summary, phylogenetic analysis using large genome databases allowed us to demonstrate that both core genome and accessory genome typing strategies grouped *S. marcescens* strains in a similar manner. Both historical and recent outbreak isolates clustered in two groups where most of the clinical strains were found. The only exception was the historical 13F-69 isolate, closer to strains with a more “environmental” style of life, probably open to high genetic exchange, including the acquisition of a complete plasmid from another environmental genus, *Bordetella* (Hamidou Soumana et al., 2017). Historical strains carried resistance genes toward antimicrobials not yet commercialized in 1969, suggesting the presence of factors driving the evolution and transmission of those genes that are not necessarily related to the antibiotic therapy. Finally, our historical isolates appeared to show a certain evolutionary dynamism during their resting period, although much lower than previously described for *E. coli.*

As shown in this study, WGS is becoming a critical tool for bacterial typing, permitting the analysis of core and accessory genomes with powerful discrimination. Even more importantly, WGS enlarges our understanding of the phylogenetic history of our outbreak isolates, offering an evolutionary perspective that will be useful for developing focused public health interventions (Baquero et al., 2015).

## Data Availability Statement

The datasets generated for this study can be found in the BioProject PRJNA510235.

## Ethics Statement

This study was carried out in accordance with the recommendations of Spanish Legislation for Good Practices and the Ley Orgánica 3/2018 on Personal Data Protection. The protocol was approved by the Ethical Committee of the University Hospital La Paz. All subjects or their legal guardians gave written informed consent in accordance with the Declaration of Helsinki.

## Author Contributions

FB and RC conceived the experiments and supervised the final manuscript. EE, FL-P, MP, and JR provided the data or strains. CS, MP-A, BP-V, VL, and LM performed the experiments, bioinformatic analysis, and drafted the initial manuscript. All authors read and approved the final version of the manuscript.

## Conflict of Interest

The authors declare that the research was conducted in the absence of any commercial or financial relationships that could be construed as a potential conflict of interest.

## Footnotes

1 http://www.applied-maths.com/bionumerics/bionumerics.htm

2 https://github.com/tseemann/nullarbor

3 https://github.com/tseemann/abricate

4 https://github.com/tseemann/snippy

5 https://www.ncbi.nlm.nih.gov/refseq/

6 www.eucast.org

## Supplementary Material

DATA SHEET S1 Metadata of the 452 genomes of Serratia marcescens available in the database of the National Center for Biotechnology Information (NCBI) RefSeq that have been used in this work.
